# The Role of Vitamins in Preconception Care and Infertility Treatment: A Narrative Review

**DOI:** 10.1002/rmb2.70068

**Published:** 2026-06-12

**Authors:** Kuniaki Ota, Toshifumi Takahashi

**Affiliations:** ^1^ Department of Obstetrics and Gynecology Kawasaki Medical School Okayama Japan; ^2^ Fukushima Medical Center for Children and Women Fukushima Medical University Fukushima Japan

**Keywords:** assisted, folic acid, infertility, preconception care, reproductive techniques, vitamin D

## Abstract

**Background:**

Micronutrient supplementation is widely used in infertility care and preconception management, yet clinical evidence supporting its effectiveness differs among vitamins.

**Methods:**

This narrative review summarizes current evidence on water‐ and fat‐soluble vitamins in women undergoing infertility treatment, with an emphasis on assisted reproductive technology (ART) outcomes, biological mechanisms, and clinical relevance from a preconception care perspective.

**Main Findings:**

Among water‐soluble vitamins, folate and vitamin B12 show the most consistent associations with ART outcomes, reflecting their central roles in one‐carbon metabolism, DNA synthesis, and methylation. Genetic variation in folate metabolism, including methylenetetrahydrofolate reductase polymorphisms, may further modify reproductive outcomes and influence folate formulation choice. In contrast, clinical evidence for vitamins B6 and C remains limited despite biological plausibility. Vitamin D deficiency is highly prevalent among infertile women and has been associated with less favorable reproductive outcomes, although supplementation trials suggest modest and context‐dependent benefits. Evidence supporting vitamin E or vitamin A use is largely confined to surrogate markers or specific phenotypes.

**Conclusion:**

Despite biological plausibility and widespread use, current evidence does not support routine vitamin supplementation in infertility care. A targeted approach based on documented deficiencies and clinical phenotype is more appropriate.

## 1 Introduction

Preconception care has evolved from a pregnancy‐centered concept to a broader approach aimed at improving maternal health and reproductive outcomes through interventions before conception [1]. Nutrition is a central component of preconception care. Among micronutrients, folate has an established role; the US Preventive Services Task Force recommends daily supplementation with 400–800 μg of folate starting at least 1 month prior to conception to reduce the risk of neural tube defects [2]. In addition to folate, accumulating evidence suggests that other micronutrients may also influence fertility and reproductive outcomes [3].

Infertility represents a major global health concern, affecting a substantial proportion of individuals and couples of reproductive age. The World Health Organization estimates that approximately 17.5% of adults worldwide experience infertility at some point during their lives [4]. Treatment for couples struggling with infertility is, in fact, the ideal setting for implementing preconception care. Micronutrients including vitamins with antioxidants, endocrine, or one‐carbon metabolic functions have gained attention as potential adjuncts to infertility treatment. In women undergoing infertility treatment, particularly assisted reproductive technology (ART), attention has expanded to a wider range of vitamins that may affect ovarian function, embryo competence, endometrial receptivity, and implantation. Although micronutrient supplementation is widely used in fertility care, recent evidence syntheses indicate substantial heterogeneity in effects across infertility phenotypes, study designs, and reproductive outcomes, with overall certainty of evidence remaining limited [3].

This narrative review summarizes the current evidence on water‐ and fat‐soluble vitamins in women undergoing infertility treatment from a preconception care perspective. The focus is on outcomes related to ART, mechanistic insights, and clinical relevance. A summary of the biological class, main evidence signal, and relative strength of evidence for each vitamin is provided in Table 1.

**TABLE 1:** Summary of evidence signals and relative strength of evidence for vitamins in reproductive medicine.

Vitamin	Class	Main evidence signal	Relative strength of evidence
Vitamin D	Fat‐soluble	Strong mechanistic plausibility; observational ART associations; couples‐based live birth data; PCOS‐related benefit; high prevalence of deficiency in infertility care	Higher
Folate (Vitamin B9)	Water‐soluble	Established role in preconception care; one‐carbon metabolism; observational associations with ART and live birth	Higher
Vitamin B12	Water‐soluble	Folate–B12–homocysteine axis; threshold association with IVF‐ET pregnancy; relevance to repeated implantation failure	Moderate
Vitamin E	Fat‐soluble	Antioxidant effects; improvement in endometrial thickness and uterine perfusion; limited evidence for hard reproductive outcomes	Moderate
Vitamin C	Water‐soluble	Oxidative stress‐related rationale; limited evidence for improvement in ART outcomes	Lower
Vitamin A	Fat‐soluble	Strong mechanistic role in retinoic acid signaling, decidualization, and embryo competence; limited direct ART intervention data	Lower
Vitamin B6	Water‐soluble	Biologic role in transsulfuration and homocysteine metabolism; limited direct reproductive evidence	Lower

Abbreviations: ART, assisted reproductive technology; IVF‐ET, in vitro fertilization and embryo transfer; PCOS, polycystic ovary syndrome.

## 2 Methods of Literature Review

This narrative review was based on a targeted literature search of PubMed/MEDLINE and Google Scholar, supplemented by manual screening of reference lists from relevant reviews, meta‐analyses, clinical studies, and guidelines. The search focused on studies published up to May 2026 and used combinations of the following terms: “vitamin,” “folate,” “folic acid,” “5‐methyltetrahydrofolate,” “vitamin B12,” “vitamin B6,” “vitamin C,” “vitamin D,” “vitamin E,” “vitamin A,” “homocysteine,” “methylenetetrahydrofolate reductase (MTHFR),” “infertility,” “assisted reproductive technology (ART),” “in vitro fertilization (IVF),” “intracytoplasmic sperm injection (ICSI),” “implantation,” “clinical pregnancy,” “live birth,” “polycystic ovary syndrome (PCOS),” and “recurrent pregnancy loss.”

Priority was given to systematic reviews, meta‐analyses, randomized controlled trials, large prospective or retrospective cohort studies, and clinically relevant mechanistic studies. Observational studies were included when they provided evidence regarding reproductive outcomes, biomarker status, or biologically plausible mechanisms relevant to infertility treatment. Studies focusing exclusively on pregnancy complications after conception, animal experiments without clear relevance to human reproductive medicine, and articles without accessible full text or sufficient methodological detail were generally excluded. When conflicting findings were identified, interpretation was guided by study design, sample size, clinically relevant endpoints such as live birth and clinical pregnancy, and consistency with existing systematic reviews.

## 3 Water‐Soluble Vitamins

Water‐soluble vitamins are relevant to reproductive medicine because of their roles in one‐carbon metabolism, nucleotide synthesis, methylation, mitochondrial function, and antioxidant defense—processes essential for normal gametogenesis, embryo development, and implantation. Among these, folate and vitamin B12 are of greatest clinical interest in women undergoing infertility treatment, as they are biochemically linked and pretreatment circulating levels have been associated with ART outcomes. In contrast, vitamin B6 and vitamin C have plausible mechanistic roles, but clinical evidence remains limited and inconsistent.

### 3.1 Folate

Folate is the naturally occurring form of vitamin B9 found in foods and present in cells as reduced tetrahydrofolate derivatives directly involved in one‐carbon metabolism. In contrast, folic acid is a fully oxidized, synthetic form used in supplements and food fortification. Folic acid requires enzymatic reduction and conversion to the biologically active folate forms before entering cellular pathways [5]. Both support DNA synthesis and methylation, but their metabolism and bioavailability differ [6].

#### 3.1.1 Clinical Evidence for Folate Intake in Women Undergoing ART

Folate intake in women undergoing ART is associated with improved reproductive outcomes, extending its role beyond fetal neural tube defect prevention [2].

Folate is essential for purine and thymidylate synthesis, methyl donor generation, and DNA replication, all of which are required in rapidly proliferating cells such as granulosa cells, preimplantation embryos, and the proliferative endometrium. In the prospective EARTH cohort including 100 women undergoing 154 ART cycles, those in the highest quartile of serum folate (> 26.3 ng/mL) had a 1.62‐fold higher probability of live birth compared with women in the lowest quartile (< 16.6 ng/mL) (95% confidence interval [CI], 0.99–2.65) [7]. This association was subsequently confirmed in a larger dietary analysis of the EARTH cohort, including 232 women and 353 initiated ART cycles [8]. In this study, higher total folate intake, expressed as dietary folate equivalents, was positively associated with implantation, clinical pregnancy, and live birth. The adjusted live birth rates across increasing quartiles of total folate intake were 30%, 47%, 42%, and 56%, respectively (P‐trend = 0.01) [8]. When supplemental folate was considered separately, women consuming > 800 μg/day had a 20% higher live birth rate than those consuming < 400 μg/day. Higher supplemental folate intake was also associated with higher fertilization rates and a lower probability of cycle failure before embryo transfer (P‐trend = 0.03 and 0.02, respectively) [8].

Together, these findings suggest an association between higher maternal folate status and improved ART outcomes, including live birth. However, these data are derived primarily from observational cohorts and cannot establish causality. Residual confounding by overall dietary quality, supplement use, socioeconomic status, body mass index (BMI), lifestyle factors, and infertility phenotype may partly explain these associations. Therefore, these findings should be interpreted as supportive of biological plausibility and hypothesis generation rather than definitive evidence that higher folate intake directly improves ART success.

#### 3.1.2 Paternal Folate Supplementation and Male‐Factor Infertility

Emerging evidence suggests that paternal folate supplementation may influence ART outcomes through effects on sperm genomic integrity rather than conventional semen parameters.

In the multicenter, randomized, double‐blind, placebo‐controlled FOLFIV trial, 162 couples undergoing IVF/ICSI for male‐factor infertility were randomized to receive either 15 mg/day folic acid or placebo for 3 months before semen collection [9]. Biochemical pregnancy after fresh embryo transfer was significantly higher in the folic acid group than in the placebo group (44.1% vs. 22.4%, *p* = 0.01), and clinical pregnancy showed a nonsignificant upward trend (35.6% vs. 20.4%, *p* = 0.08). Although conventional semen parameters did not materially change, sperm DNA fragmentation decreased significantly in the folic acid arm (8.5 ± 4.5 vs. 6.4 ± 4.6, *p* < 0.0001).

These data suggest that paternal folate status may contribute to reproductive success by improving sperm DNA integrity rather than classical semen quality, highlighting a potential but still limited role for male‐targeted folate supplementation in ART.

#### 3.1.3 Folate Metabolic Pathways

**FIGURE 1:**
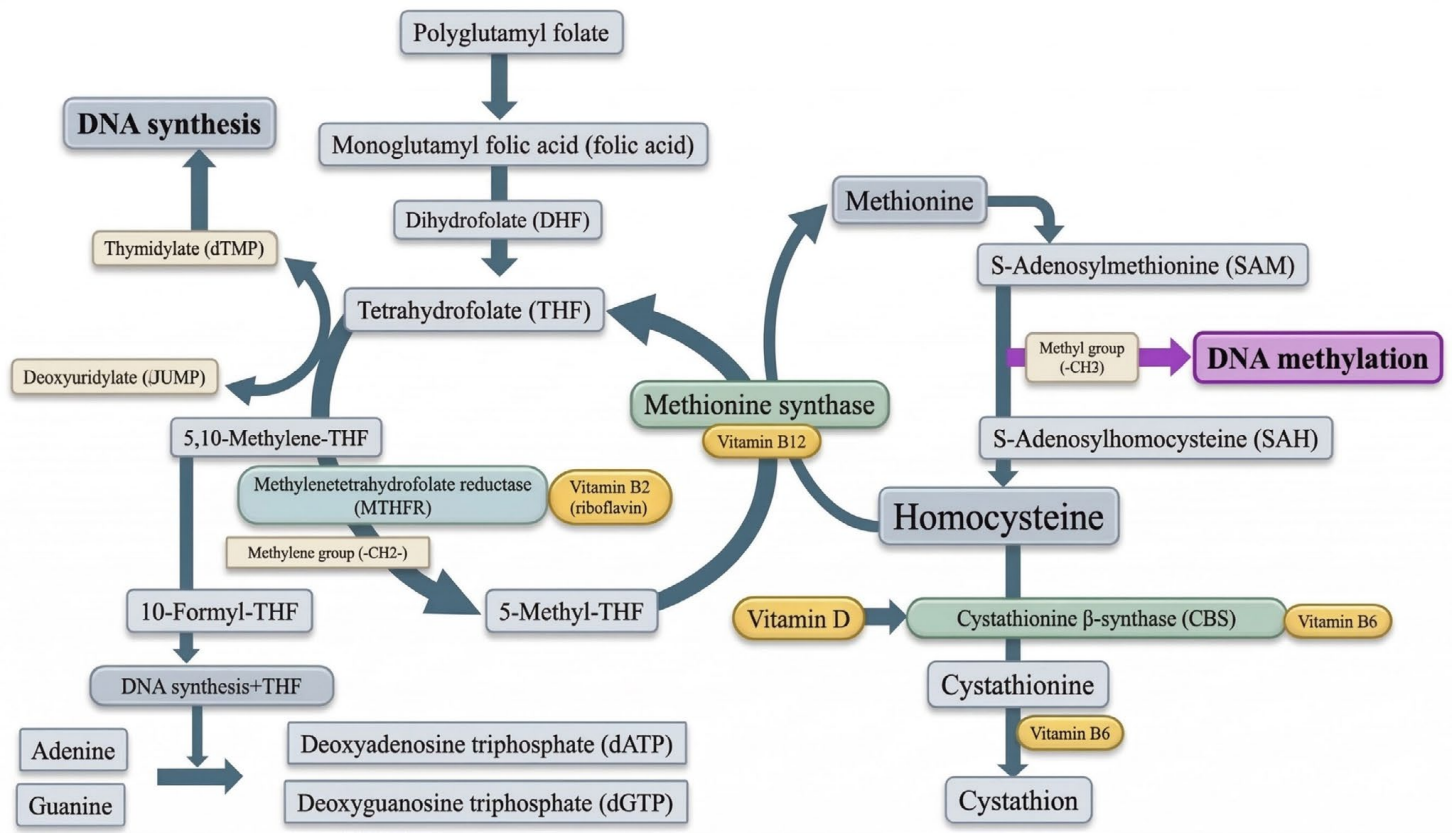
Folate‐dependent one‐carbon metabolism and proposed interactions with vitamin D in infertility treatment. Dietary folate and supplemental folic acid are converted to tetrahydrofolate (THF) derivatives that participate in one‐carbon metabolism. 5,10‐Methylene‐THF contributes to thymidylate synthesis, whereas 10‐formyl‐THF supports purine synthesis. 5‐Methyl‐THF donates a methyl group for the remethylation of homocysteine to methionine through vitamin B12‐dependent methionine synthase, thereby contributing to the generation of S‐adenosylmethionine (SAM), the principal methyl donor for DNA methylation. Homocysteine can also enter the transsulfuration pathway via vitamin B6‐dependent cystathionine β‐synthase (CBS) to form cystathionine. In addition, active vitamin D signaling may directly induce CBS expression, thereby promoting homocysteine disposal through transsulfuration. Disruption of these integrated pathways may impair nucleotide synthesis, epigenetic regulation, redox balance, and implantation‐related processes.

To clarify the potential benefits of folate for fertility, an understanding of one‐carbon metabolism is essential (Figure 1). In this pathway, tetrahydrofolate (THF) derivatives derived from dietary folate serve two key biological functions. First, 5,10‐methylene‐THF and 10‐formyl‐THF support thymidylate and purine synthesis, thereby enabling DNA replication in rapidly proliferating tissues. Second, 5‐methyl‐THF donates a methyl group for the remethylation of homocysteine to methionine via vitamin B12–dependent methionine synthase. Methionine is subsequently converted to S‐adenosylmethionine, the universal methyl donor required for DNA methylation and other epigenetic processes. Insufficient folate availability may therefore compromise both nucleotide synthesis and methylation capacity, providing a biologically plausible mechanism linking folate status to fertility and early developmental processes [10, 11].

Within this metabolic network, methylenetetrahydrofolate reductase (MTHFR) plays a central regulatory role by catalyzing the irreversible conversion of 5,10‐methylene‐THF to 5‐methyl‐THF, the biologically active form required for homocysteine remethylation [12]. Through this reaction, MTHFR directly influences one‐carbon flux, methylation potential, and nucleotide metabolism. Reduced MTHFR activity can result in impaired methylation capacity and elevated homocysteine levels, further supporting the importance of folate metabolism in reproductive biology [13].

#### 3.1.4 MTHFR Gene Polymorphism in Reproductive Medicine

MTHFR gene polymorphisms have been associated with altered gamete and embryo quality in women undergoing ART, suggesting a potential impact of folate‐dependent one‐carbon metabolism on reproductive outcomes.

In a retrospective cohort of 1160 women undergoing in vitro fertilization (IVF) or intracytoplasmic sperm injection (ICSI), oocyte maturation declined in a dose‐dependent manner with decreasing predicted MTHFR activity based on combined C677T/A1298C genotypes; women with the lowest‐activity genotype (677TT/1298AA) showed significantly reduced maturation rates, while embryo quality was less favorable in those with intermediate activity [14]. Similarly, in a cohort of 1173 women undergoing a first IVF/ICSI cycle, the MTHFR 677TT genotype was associated with fewer transferable and good‐quality embryos and a lower cumulative live birth rate among women treated with a gonadotropin releasing hormone agonist short protocol, although first‐transfer clinical pregnancy and live birth rates were not significantly different [15].

Together, these findings suggest that MTHFR polymorphisms are linked to impaired oocyte maturation and embryo quality, potentially influencing cumulative ART success through disrupted folate metabolism rather than immediate implantation or early pregnancy outcomes.

#### 3.1.5 Optimal Folate Formulation in the Presence of MTHFR Variants

An important practical question in reproductive medicine is whether conventional folic acid is always the optimal folate formulation, particularly in couples carrying MTHFR polymorphisms that impair one‐carbon metabolism.

As noted in a 2022 review by Menezo and colleagues, synthetic folic acid requires reduction by dihydrofolate reductase followed by processing through the folate cycle to become biologically active. In individuals with polymorphisms affecting one‐carbon metabolism, high‐dose folic acid may therefore be inefficiently metabolized, resulting in less increased active folate availability and more in accumulation of unmetabolized folic acid (UMFA) [16]. In contrast, L‐5‐methyl‐THF represents the predominant circulating and biologically active folate form delivered to peripheral tissues. A review by Pietrzik et al. reported that L‐5‐methyl‐THF exhibits physiologic activity and bioavailability comparable to folic acid; moreover, earlier utilization studies cited in that review demonstrated that peak plasma folate concentrations following 5‐methyl‐THF administration were nearly sevenfold higher than those observed after folic acid [17].

Clinical data further suggest differential metabolic consequences of these formulations. In a recent feasibility randomized controlled trial, 22 couples with recurrent pregnancy loss were assigned to prenatal multivitamins containing either 5‐methyl‐THF or folic acid. UMFA concentrations decreased in the 5‐methyl‐THF group but increased in the folic acid group, with mean follow‐up levels of approximately 0.39 and 2.78 nmol/L, respectively [18]. Importantly, because MTHFR polymorphisms such as C677T reduce the efficiency of converting folic acid to 5‐methyl‐THF, L‐5‐methyl‐THF can be utilized directly without MTHFR‐dependent activation, thereby bypassing a potential metabolic bottleneck.

Collectively, these findings suggest that while folic acid remains effective in the general population, L‐5‐methyl‐THF may offer metabolic advantages in individuals with impaired folate activation, supporting consideration of genotype‐informed folate formulation selection in reproductive disorders.

### 3.2 Vitamin B12

Vitamin B12 (cobalamin) functions intracellularly as an essential coenzyme in two key metabolic reactions. In the cytosol, methylcobalamin serves as a cofactor for methionine synthase, which catalyzes the transfer of a methyl group from 5‐methyl THF to homocysteine, thereby regenerating tetrahydrofolate and producing methionine. This reaction supports de novo DNA synthesis and cellular methylation through the generation of S‐adenosylmethionine [19]. In mitochondria, adenosylcobalamin serves as a cofactor for methylmalonyl‐CoA mutase, catalyzing the conversion of methylmalonyl‐CoA to succinyl‐CoA. Vitamin B12 deficiency is associated with neurologic dysfunction and impaired myelin integrity [20].

#### 3.2.1 Clinical Evidence for Serum Vitamin B12 Levels in Reproductive Medicine

Serum vitamin B12 status has been positively associated with ART outcomes, particularly live‐birth rates, suggesting a supportive role of adequate cobalamin availability in human reproduction.

In the EARTH cohort, women in the highest quartile of serum vitamin B12 (> 701 pg/mL) had 2.04 times the probability of live birth compared with those in the lowest quartile (< 439 pg/mL) (95% CI, 1.14–3.62) [7]. Moreover, women with both serum folate and vitamin B12 concentrations above the median had 1.92 times the probability of live birth (95% CI, 1.12–3.29), corresponding to an adjusted absolute increase in live birth rate of 26% (95% CI, 10%–48), compared with women in whom both were at or below the median [7]. In a retrospective study of 356 women undergoing IVF‐ET, serum vitamin B12 was nonlinearly associated with clinical pregnancy. Specifically, when serum vitamin B12 was ≤ 358.7 pg/mL, each 10 pg/mL increase was associated with a 4% increase in clinical pregnancy rate (adjusted odds ratio [aOR] 1.04, 95% CI 1.00–1.08), whereas no clear additional benefit was observed above this threshold [21].

Collectively, these data indicate that adequate vitamin B12 status is associated with improved ART outcomes, particularly in women with low or insufficient baseline levels, supporting a threshold‐dependent role of vitamin B12 within folate‐dependent one‐carbon metabolism in reproductive medicine.

#### 3.2.2 Clinical Evidence Linking Serum Homocysteine to the Folate–Vitamin B12 Pathway in Reproductive Medicine

Homocysteine represents a key metabolic node linking folate and vitamin B12–dependent one‐carbon metabolism, but its direct association with ART outcomes remains clinically inconsistent.

Homocysteine is a sulfur‐containing, non‐proteinogenic amino acid that serves as a key metabolic intermediate linking methionine, folate, and vitamin B12 metabolism; elevated levels may contribute to oxidative stress, endothelial dysfunction, and impaired cellular function. Clinical evidence linking serum homocysteine to IVF outcomes remains inconsistent. A retrospective study published in 2023 reported that higher serum homocysteine levels were positively correlated with the number of repeated implantation failures and cumulative failed embryo transfers, even though the majority of patients (95.8%) had homocysteine concentrations within the conventionally normal range (< 14 μmol/L), suggesting a potential subclinical association [22] Earlier work from 2007 proposed that hyperhomocysteinemia might adversely affect pregnancy rate, implantation rate, and miscarriage risk in women undergoing IVF [23].

In contrast, a prospective study published in 2018 found that higher folate concentrations, rather than homocysteine itself, were associated with improved clinical pregnancy rates following IVF, with no strong independent association observed between serum homocysteine levels and reproductive outcomes [24]. Similarly, a 2019 case–control study concluded that serum homocysteine levels were not significantly associated with IVF or ICSI outcomes [25].

Taken together, current evidence suggests that homocysteine reflects disturbances in folate–vitamin B12 metabolism, but it should not be regarded as a robust independent or causal predictor of ART outcomes. Observed associations between homocysteine and implantation failure or IVF outcomes may be confounded by nutritional status, renal function, inflammatory conditions, metabolic disease, lifestyle factors, and underlying infertility phenotype. Homocysteine may therefore be best interpreted as a downstream or integrative marker of broader one‐carbon metabolic imbalance rather than a direct causal determinant of reproductive success.

### 3.3 Vitamin B6

Vitamin B6 is a water‐soluble vitamin that exists in several interconvertible forms, including pyridoxine, pyridoxal, and pyridoxamine, with pyridoxal 5′‐phosphate serving as the biologically active coenzyme form. It acts as a cofactor in amino acid metabolism and sulfur amino acid metabolism [26]. In homocysteine‐related pathways, vitamin B6 is particularly important for transsulfuration, in which homocysteine is converted to cystathionine through cystathionine β‐synthase, thereby contributing to redox homeostasis and sulfur metabolism [27] (Figure 1).

#### 3.3.1 Clinical Evidence for Vitamin B6 in Reproductive Medicine

Despite its biologically plausible role within one‐carbon and amino acid metabolism, direct clinical evidence supporting a beneficial effect of vitamin B6 on infertility or ART outcomes remains limited.

In a prospective IVF‐ET study, no significant association was observed between maternal serum vitamin B6 status and implantation, clinical pregnancy, live birth, or early miscarriage, whereas more consistent associations were seen for riboflavin, folate, and vitamin B12‐related markers [28]. This is consistent with the recent umbrella review, which found no convincing evidence that vitamin B‐complex supplementation improves clinical pregnancy or live birth in female infertility, although the certainty of evidence was very low [3].

Taken together, current data suggest that while vitamin B6 is metabolically important, its isolated contribution to reproductive outcomes appears limited, and available clinical evidence does not support vitamin B6 supplementation as an effective stand‐alone intervention for enhancing fertility or ART success.

### 3.4 Vitamin C

Vitamin C (ascorbic acid) is a water‐soluble vitamin that functions as a major nonenzymatic antioxidant and as a cofactor in several enzymatic reactions, including collagen synthesis and cellular redox regulation [29]. In reproductive tissues, vitamin C may help protect cells from oxidative damage by scavenging reactive oxygen species and supporting antioxidant defense systems. Because oxidative stress has been implicated in endometriosis, oocyte dysfunction, embryo–endometrial interaction during ART, vitamin C has been considered a biologically plausible adjunct in reproductive medicine [30, 31].

#### 3.4.1 Clinical Evidence for Vitamin C in Reproductive Medicine

Despite strong biological plausibility based on its antioxidant properties, current clinical evidence does not support a clear reproductive benefit of vitamin C supplementation in infertility treatment or ART outcomes.

To date, only a small number of studies have investigated the impact of vitamin C administration during human IVF–ET cycles. In a randomized study of women with endometriosis undergoing IVF‐ET, 2 months of oral vitamin C supplementation at 1000 mg/day improved serum and follicular‐fluid vitamin C concentrations but did not significantly improve oxidative stress markers or reproductive outcomes [30]. Implantation rates were 28.0% (65/232) in the treated group versus 23.1% (45/195) in the untreated endometriosis group, and clinical pregnancy rates were 39.4% (54/137) versus 33.3% (36/108), respectively; these differences were not statistically significant [30]. This is consistent with the recent umbrella review, which found no reliable evidence that vitamin C supplementation improves clinical pregnancy rates in women with infertility [3].

Collectively, available data suggest that although vitamin C supplementation can effectively increase systemic and follicular antioxidant levels, this biochemical effect does not translate into demonstrable improvements in implantation, clinical pregnancy, or ART success, limiting its current role as a fertility‐enhancing intervention.

## 4 Fat‐Soluble Vitamins

### 4.1 Vitamin D

Vitamin D is a fat‐soluble vitamin that is synthesized in the skin under ultraviolet B exposure or obtained from dietary sources, and is subsequently converted in the liver to 25‐hydroxyvitamin D (25(OH)D) and in the kidney and other tissues to its active form, 1,25‐dihydroxyvitamin D_3_. Beyond its classical role in calcium and bone metabolism, vitamin D is involved in cellular differentiation, immune modulation, and endocrine regulation [32]. In reproductive medicine, vitamin D receptor expression and vitamin D‐metabolizing enzymes have been demonstrated in the ovary, endometrium, and placenta, supporting a potential role in folliculogenesis, steroidogenesis, endometrial receptivity, decidualization, and implantation [33].

#### 4.1.1 Prevalence of Vitamin D Deficiency in Infertile Women

Globally, vitamin D status is most assessed using serum 25(OH)D concentrations. In epidemiological and reproductive studies, levels < 20 ng/mL (50 nmol/L) are typically defined as deficient, 20–29 ng/mL (50–75 nmol/L) as insufficient, and ≥ 30 ng/mL (≥ 75 nmol/L) as sufficient. However, recent Endocrine Society guideline communications have emphasized ongoing uncertainty regarding optimal cutoffs, particularly for nonskeletal outcomes, and no single serum 25(OH)D threshold has been definitively associated with universal clinical benefit [34].

At the global level, vitamin D deficiency is highly prevalent among women of reproductive age. A pooled analysis of population‐based studies including more than 7.9 million participants from 81 countries reported that approximately 47.9% of individuals had serum 25(OH)D levels < 20 ng/mL and 76.6% had levels < 30 ng/mL, with consistently higher rates of deficiency observed in women than in men [35].

This pattern extends to infertile populations. A large European cross‐sectional study of 1072 infertile women attending assisted reproduction centers in Italy found that 40.1% had serum 25(OH)D levels of ≤ 20 ng/mL, and 77.4% had levels of ≤ 30 ng/mL throughout the year. These results indicate a high prevalence of vitamin D deficiency among women seeking infertility care [36]. Similar findings have been reported in Northern Europe. In Sweden, approximately 27% of women undergoing IVF treatment were vitamin D insufficient (< 50 nmol/L [20 ng/mL]), with a substantially higher prevalence observed in women from non‐Nordic regions compared to those from Nordic countries [37].

Among available data on vitamin D status in Asian women with infertility, a report from Japan is noteworthy because of its large sample size. Real‐world data from Japan further underscore the clinical relevance of vitamin D deficiency in infertility care. In a 2026 multicenter cross‐sectional study of 17 261 women attending infertility clinics, the median serum 25(OH)D concentration was 16.4 ng/mL; 66.8% of women were vitamin D deficient, and 89.0% were insufficient [38].

#### 4.1.2 Clinical Evidence for Vitamin D in Reproductive Medicine

Clinical evidence from observational and interventional studies suggests a possible association between vitamin D status and reproductive outcomes, although the magnitude and consistency of benefit vary by study design, baseline vitamin D status, and supplementation strategy.

Observational evidence in ART consistently suggests that vitamin D sufficiency is associated with improved reproductive outcomes. Meta‐analyses of observational studies have shown higher odds of biochemical pregnancy, ongoing pregnancy, and live birth among vitamin D‐sufficient women, whereas deficiency is associated with lower clinical pregnancy (OR 0.81, 95% CI 0.70–0.95) and live birth rates (OR 0.69, 95% CI 0.54–0.89) [39, 40]. Population‐level observational data support these associations. In Denmark, exposure to vitamin D fortification was associated with an increased likelihood of live birth [41] and in a large prospective infertility cohort, vitamin D sufficiency was associated with higher live birth rates in women (adjusted relative ratio [aRR], 95% CI 1.05–1.56) and couples (aRR 1.26, 95% CI 1.00–1.58) [42].

Evidence from interventional studies on vitamin D supplementation in ART remains mixed and outcome‐dependent. In a trial‐sequential meta‐analysis of five randomized controlled trials restricted to vitamin D‐deficient women undergoing IVF, vitamin D supplementation increased chemical pregnancy (RR 1.53, 95% CI 1.06–2.20), but not clinical pregnancy (RR 1.34, 95% CI 0.81–2.24), and no clear benefit was demonstrated for the secondary reproductive endpoints that were available [43]. In contrast, a larger 2023 systematic review and meta‐analysis including both randomized and cohort studies (12 studies, *n* = 2352) reported a significant improvement in clinical pregnancy rates associated with vitamin D supplementation (OR 1.70, 95% CI 1.24–2.34), whereas implantation, biochemical pregnancy, miscarriage, and multiple pregnancy rates were not consistently affected [44]. Subgroup analyses in the latter review suggested that benefits were most apparent in women with baseline serum 25(OH)D levels below 30 ng/mL and with moderate daily dosing (1000–10 000 IU) administered for at least 30–60 days, rather than single high‐dose bolus regimens.

Taken together, available evidence indicates that vitamin D sufficiency is associated with more favorable reproductive outcomes in several observational studies, but causality remains uncertain. These associations may be influenced by residual confounding, including BMI, ethnicity, sunlight exposure, dietary patterns, socioeconomic status, comorbidities, infertility phenotype, and general health behaviors. Interventional evidence suggests that supplementation may have modest benefits in selected vitamin D‐deficient populations, but the magnitude and consistency of benefit remain limited. Therefore, vitamin D supplementation should be considered as a targeted correction of deficiency rather than a universally effective fertility‐enhancing intervention.

#### 4.1.3 Mechanistic Links Between Vitamin D and Homocysteine Metabolism

Experimental evidence indicates that vitamin D directly modulates homocysteine metabolism through the transsulfuration pathway. Kriebitzsch et al. showed that 1,25dihydroxyvitamin D_3_ upregulates cystathionine β‐synthase, the rate‐limiting enzyme that redirects homocysteine toward cystathionine synthesis, resulting in reduced intracellular homocysteine and increased sulfur‐containing metabolites. This mechanism provides a biologically plausible link between vitamin D status, homocysteine clearance, and redox regulation within reproductive tissues [45].

#### 4.1.4 Clinical Evidence Linking Vitamin D and Homocysteine Metabolism in Reproductive Medicine

Building on mechanistic evidence, clinical studies support an inverse association between vitamin D status and homocysteine levels in women of reproductive age, including those with infertility and pregnancy loss.

Interventional data demonstrate that vitamin D supplementation can reduce circulating homocysteine levels. In overweight women of reproductive age, weekly administration of 50 000 IU vitamin D_3_ for 2 months resulted in a significant decrease in serum homocysteine compared with placebo [46]. Consistent with these findings, in infertility‐related polycystic ovary syndrome (PCOS), serum 25(OH)D concentrations were inversely correlated with homocysteine (*r* = −0.392, *p* < 0.001) and remained an independent negative correlate after multivariable adjustment (*β* = −0.316, *p* = 0.006) [47].

Further support for this metabolic link comes from a large retrospective cross‐sectional study of 837 women with recurrent pregnancy loss, in which carriers of the MTHFR 677TT genotype exhibited significantly lower serum 25(OH) vitamin D levels and higher homocysteine concentrations compared with CC and CT genotypes. In the same cohort, vitamin D insufficiency (< 30 ng/mL) was identified as an independent risk factor for hyperhomocysteinemia (aOR 1.89, 95% CI 1.41–2.52), and a significant inverse correlation between vitamin D and homocysteine was observed specifically among TT carriers [48].

Although derived from heterogeneous reproductive populations, these findings consistently indicate that vitamin D deficiency, impaired homocysteine clearance, and MTHFR‐related one‐carbon metabolic dysfunction are biologically interconnected, extending the mechanistic link into clinically observable metabolic phenotypes.

#### 4.1.5 Vitamin D Supplementation and Reproductive Outcomes in Women With PCOS

Beyond metabolic associations, clinical outcome data suggest that vitamin D supplementation in women with PCOS may preferentially affect pregnancy maintenance rather than early gamete or embryo‐level processes.

In women with PCOS, a systematic review and meta‐analysis reported that vitamin D supplementation was associated with higher pregnancy rates among women with baseline vitamin D insufficiency (RR 1.57, 95% CI 1.29–1.90) and a significantly reduced risk of early miscarriage (RR 0.44, 95% CI 0.30–0.66). In contrast, fertilization and embryonic cleavage rates were not significantly improved, indicating a lack of effect on early embryologic development [49].

### 4.2 Vitamin E

Vitamin E is a fat‐soluble vitamin that consists of tocopherols and tocotrienols, of which α‐tocopherol is the predominant biologically active form in humans. As a lipid‐soluble antioxidant, vitamin E interrupts lipid peroxidation within cellular membranes and helps protect cells from oxidative damage. In reproductive tissues, this antioxidant property is biologically relevant because oxidative stress has been implicated in granulosa‐cell dysfunction, impaired endometrial development, and embryo–endometrial interaction [50, 51].

#### 4.2.1 Clinical Evidence for Vitamin E in Reproductive Medicine

Vitamin E has been studied in reproductive medicine mainly for its antioxidant properties and potential effects on endometrial development; however, evidence for improvements in clinically decisive reproductive outcomes remains limited.

In a 2021 systematic review and meta‐analysis of seven clinical trials including 652 infertile women, vitamin E supplementation significantly increased endometrial thickness (standardized mean difference 0.57, 95% CI 0.26–0.87), but it did not significantly improve ongoing pregnancy rates (OR 1.08, 95% CI 0.72–1.62) [52]. Individual clinical studies are broadly consistent with these findings. Cicek et al. reported that, in women with unexplained infertility undergoing controlled ovarian stimulation, endometrial thickness on the day of human chorionic gonadotropin administration was significantly greater in the vitamin E group than in controls (9.6 ± 2.1 vs. 8.2 ± 2.0 mm; *p* = 0.001). In contrast, neither implantation rates (18.9% vs. 16.0%) nor ongoing pregnancy rates (18.9% vs. 14.0%) differed significantly between groups [53]. Takasaki et al. demonstrated that, in women with thin endometrium and elevated uterine radial artery resistance, vitamin E supplementation improved radial artery resistance in 72% and endometrial thickness in 52% of treated patients, accompanied by histological evidence of enhanced glandular epithelial growth, vascular development, and increased VEGF expression [54]. Consistent with findings in non‐PCOS populations, evidence in PCOS infertile women remains limited. In a randomized open‐label trial of women with clomiphene citrate–resistant PCOS, Morsy et al. reported that adjunctive vitamin E supplementation (1500 IU/day) added to metformin plus clomiphene did not significantly improve ovulation or pregnancy rates, although endometrial thickness was significantly greater in the vitamin E group [55].

Taken together, the available evidence suggests that vitamin E is more relevant to endometrial support than to direct improvements in pregnancy or live birth outcomes. Current data therefore support its biological plausibility in selected endometrial phenotypes, but not its routine use as a fertility‐enhancing intervention.

### 4.3 Vitamin A

Vitamin A is a fat‐soluble micronutrient that includes retinol, retinal, retinyl esters, and biologically active retinoic acid derivatives. Retinoic acid regulates gene expression through retinoic acid receptors and retinoid X receptors and is essential for growth, differentiation, and tissue development [56]. In female reproductive tissues, retinoid signaling has been implicated in key processes including folliculogenesis, steroidogenesis, uterine receptivity, decidualization, and early placentation, primarily based on findings from animal models and experimental studies [57, 58, 59].

#### 4.3.1 Evidence of the Role of Vitamin A in Reproductive Medicine

Human evidence regarding vitamin A–derived retinoids in reproductive medicine is scarce and largely limited to observational associations with follicular and embryo‐related characteristics.

In a study of 79 women undergoing IVF, Pauli et al. analyzed retinoid concentrations in peripheral plasma and follicular fluid and evaluated their association with embryo quality [60]. Follicles that yielded grade I embryos exhibited higher mean all‐trans retinoic acid concentrations in follicular fluid than those yielding nongrade I embryos, independent of follicle size. In addition, larger follicles showed more than 50% higher follicular‐fluid levels of retinol and all‐trans retinoic acid. Women with endometriosis exhibited approximately 50% lower mean all‐trans retinoic acid concentrations in both follicular fluid and plasma compared with controls [60].

## 5 Evidence for Combination Vitamin Supplementation

Combination vitamin supplementation is frequently used in infertility care based on the premise that multiple vitamins may simultaneously target complementary biological pathways involved in reproduction; however, clinical evidence supporting this approach remains limited and heterogeneous.

The 2020 Cochrane review suggested that antioxidant regimens, many of which included vitamins, were associated with higher clinical pregnancy and live birth rates than placebo or no treatment, but the certainty of evidence was low to very low and the contribution of specific vitamin components could not be isolated [61]. A subsequent umbrella review confirmed this cautious interpretation, showing that although multi‐micronutrient and antioxidant regimens often demonstrated favorable trends, the overall certainty of evidence for vitamin‐containing interventions was predominantly low or very low due to substantial heterogeneity and risk of bias, and were therefore insufficient to recommend routine use in female infertility, including ART [3]. The most informative recent comparative synthesis is a 2025 network meta‐analysis including 30 randomized controlled trials and 3977 women with infertility. Among vitamin‐only regimens, vitamin D plus vitamin E was associated with a higher clinical pregnancy rate than placebo (RR 2.76, 95% CI 1.67–5.07), whereas vitamin D alone showed a smaller effect with low certainty (RR 1.29, 95% CI 1.10–1.52) [62]. Importantly, these estimates were derived largely from indirect comparisons, and safety data were limited.

Taken together, current evidence suggests that while certain vitamin combinations may show potential benefits in selected analyses, available data do not establish definitive superiority or clinical robustness. Given the reliance on indirect evidence, heterogeneity across trials, and limited safety reporting, routine use of vitamin‐based combination supplementation to improve ART outcomes cannot presently be recommended.

## 6 Safety Considerations in Vitamin Supplementation

Although vitamin supplementation is often perceived as safe, excessive or unnecessary supplementation may carry potential risks, particularly in the preconception period. Because supplementation may be continued during the periconceptional period and early pregnancy, clinical use should balance potential benefit with safety, dose, baseline nutritional status, and the possibility of unrecognized pregnancy.

For folate, standard preconception supplementation is well established for neural tube defect prevention [2]. Recommended doses of folic acid are generally considered safe and effective. However, folic acid is a synthetic, oxidized form of folate that must be reduced by dihydrofolate reductase and subsequently converted through the folate cycle before it can participate in one‐carbon metabolism [16, 17]. When folic acid intake exceeds the capacity of these metabolic pathways, particularly in the setting of high‐dose supplementation, multiple supplement use, food fortification, or genetic variation affecting folate metabolism, UMFA may accumulate in the circulation [16, 17, 18, 63]. UMFA has also been detected in pregnancy‐related biological compartments, including maternal blood, cord blood, and human milk, suggesting that maternal folic acid exposure can influence fetal and neonatal folate profiles [64, 65]. Although the clinical significance of UMFA remains uncertain, observational studies have raised concerns regarding potential associations between altered folate subtypes and offspring neurodevelopmental outcomes [66]. These findings should be interpreted cautiously because they do not establish causality and may be affected by residual confounding. Therefore, standard‐dose folic acid supplementation for neural tube defect prevention should be clearly distinguished from unnecessary high‐dose or prolonged folic acid supplementation in infertility care.

For vitamin D, correction of deficiency is biologically and clinically reasonable, but excessive intake may cause vitamin D toxicity, typically characterized by hypercalcemia, hypercalciuria, nephrolithiasis, and renal or systemic complications [67, 68]. Because optimal serum 25(OH)D thresholds for nonskeletal reproductive outcomes remain uncertain [34], supplementation should be guided by baseline vitamin D status, dose, duration, and established safety limits rather than by the assumption that higher levels are always beneficial.

Vitamin A requires particular caution in preconception care because excessive intake of preformed vitamin A or retinoid exposure is teratogenic [69, 70]. Although vitamin A is essential for reproductive and embryonic development, high‐dose preformed vitamin A supplementation should be avoided in women who may conceive unless there is a clear medical indication.

High‐dose antioxidant supplementation, including vitamins C and E, may also have uncertain effects. Although oxidative stress is implicated in reproductive dysfunction, reactive oxygen species also participate in physiological redox signaling during folliculogenesis, ovulation, implantation, and early embryonic development [71]. Excessive antioxidant exposure could therefore theoretically disturb these processes, and current evidence does not support routine high‐dose antioxidant supplementation for all women undergoing infertility treatment [61].

Overall, vitamin supplementation in reproductive medicine should be individualized, deficiency‐oriented, and dose‐conscious. A preconception care framework should emphasize appropriate assessment, avoidance of unnecessary high‐dose supplementation, and careful consideration of potential fetal and maternal safety.

## 7 Conclusions

Among the vitamins reviewed, folate and vitamin B12 show the most consistent associations with ART outcomes, whereas evidence for vitamins B6, C, E, and A remains limited or is confined to specific phenotypes or surrogate markers. Vitamin D deficiency is highly prevalent in infertile populations and is consistently associated with poorer reproductive outcomes, although the benefits of supplementation appear modest and strongly dependent on baseline status, dose, and clinical context.

Overall, despite strong biological plausibility and widespread clinical use, current evidence does not support routine or universal vitamin supplementation as a fertility‐enhancing intervention for all women undergoing infertility treatment. Available data instead support assessment and correction of documented deficiencies and consideration of metabolic background and clinical phenotype. Because much of the evidence is observational and potentially affected by residual confounding, further well‐designed randomized controlled trials are required to determine whether personalized vitamin interventions can meaningfully improve ART success and live‐birth outcomes.

## Funding

The authors have nothing to report.

## Conflicts of Interest

The authors declare no conflicts of interest.

## Data Availability

Data sharing not applicable to this article as no datasets were generated or analyzed during the current study.
